# Mature teratoma of conus medullaris: A case report and review of literature

**DOI:** 10.1002/ccr3.7966

**Published:** 2023-09-25

**Authors:** Masoud Eslami, Mehran Ilaghi, Erfan Shahabinejad, Forouzande Khajepour, Saeed Karamouzian, Hamed Reihani‐Kermani

**Affiliations:** ^1^ Department of Neurosurgery Kerman University of Medical Sciences Kerman Iran; ^2^ Institute of Neuropharmacology, Kerman Neuroscience Research Center, Kerman University of Medical Sciences Kerman Iran; ^3^ Student Research Committee Rafsanjan University of Medical Sciences Rafsanjan Iran; ^4^ USERN Office, Rafsanjan University of Medical Sciences Rafsanjan Iran

**Keywords:** conus medullaris, mature teratoma, spinal tumor

## Abstract

In conus medullaris, mature teratomas are rare. We report a case of a 40‐year‐old man who presented with urinary incontinence, low back pain, and muscle weakness. Magnetic resonance imaging revealed a mass in conus medullaris (T_11_–L_1_), further confirmed as a mature teratoma by pathological examination. We identified 63 cases of conus medullaris teratoma over the past two decades by systematically analyzing the case reports. Findings demonstrated that most cases were diagnosed in the fourth decade of life, with the majority of cases (57.6%) being male. Lower back pain, radiating pain in the extremities, hypoesthesia, and urinary dysfunction are the most common clinical presentations among patients with teratoma of conus medullaris. Mature teratoma is the dominant pathologic subtype of teratomas in this region, comprising more than 95% of cases. Our case highlights the importance of considering spinal teratoma as a differential diagnosis in patients presenting with urinary incontinence and lumbar pain.

## Graphical abstract

Teratomas of the spinal cord are rare. We present a mature teratoma of conus medullaris (T11‐L1) presenting with prolonged symptoms.

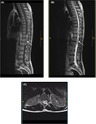

## 1 INTRODUCTION

Teratomas are generally referred to as types of germ cell tumors derived from all three germ layers, including endoderm, mesoderm, and ectoderm. While these tumors may manifest in various regions, teratomas constitute less than 1% of the central nervous system (CNS) tumors and are extremely rare in the spinal cord.^1^ In terms of pathologic subtypes, teratomas are generally divided into three categories, namely mature, immature, and malignant teratoma.^2^ Among the teratomas presenting in the spinal cord, mature teratoma of the conus medullaris is a relatively rare tumor and is less frequently reported compared to other types. In the current study, we report a rare case of conus medullaris mature teratoma presenting with prolonged symptoms and provide a review of the literature and current evidence of conus teratomas.

## 2 CASE PRESENTATION

A 40‐year‐old man was referred to our center, the main referral neurosurgery center in Southeast Iran, complaining of progressive radicular pain. The patient had a history of urinary incontinence since the age of 10. Refractory lumbar pain radiating to both lower limbs developed about 1 year before the current admission. The pain intensified with activity and was relieved when the patient was relaxed. No history of neurogenic claudication was present. Four months before the admission, the patient developed a left foot drop. At the time of admission, hypoesthesia of both lower limbs and anesthesia of the left foot were present. Moreover, saddle hypoesthesia and urinary incontinence were noted.

Grading of muscle strength revealed that the distal force of the left lower limb was significantly reduced, where the proximal and distal forces were 4/5 and 0/5, respectively. Examination of the right lower limb demonstrated that the proximal and distal force were 5/5 and 1/5, respectively. The straight leg raise (SLR) test and Patrick's test were negative. No signs of Hoffmann's reflex, Babinski reflex, or clonus were observed, and the proprioception was intact. However, the knee and ankle reflexes of both sides were absent.

**FIGURE 1:**
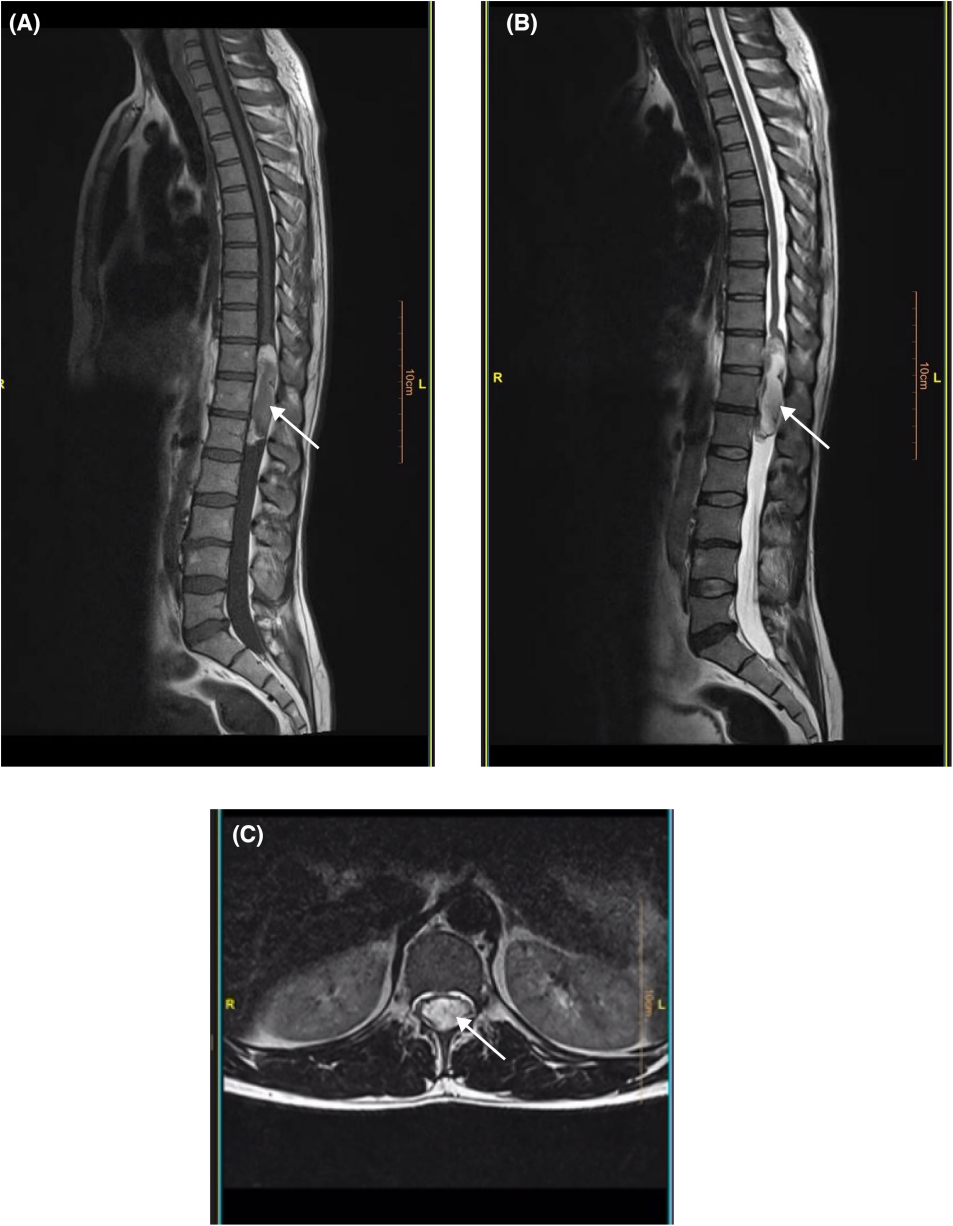
Preoperative sagittal T1‐weighted (A) and T2‐weighted (B) magnetic resonance imaging demonstrate an intradural extramedullary mass lesion at T_11_–L_1_ (conus medullaris). Axial section (C) demonstrates a large‐sized capsular lesion causing spinal cord compression. White arrows point to the tumor location.

**FIGURE 2:**
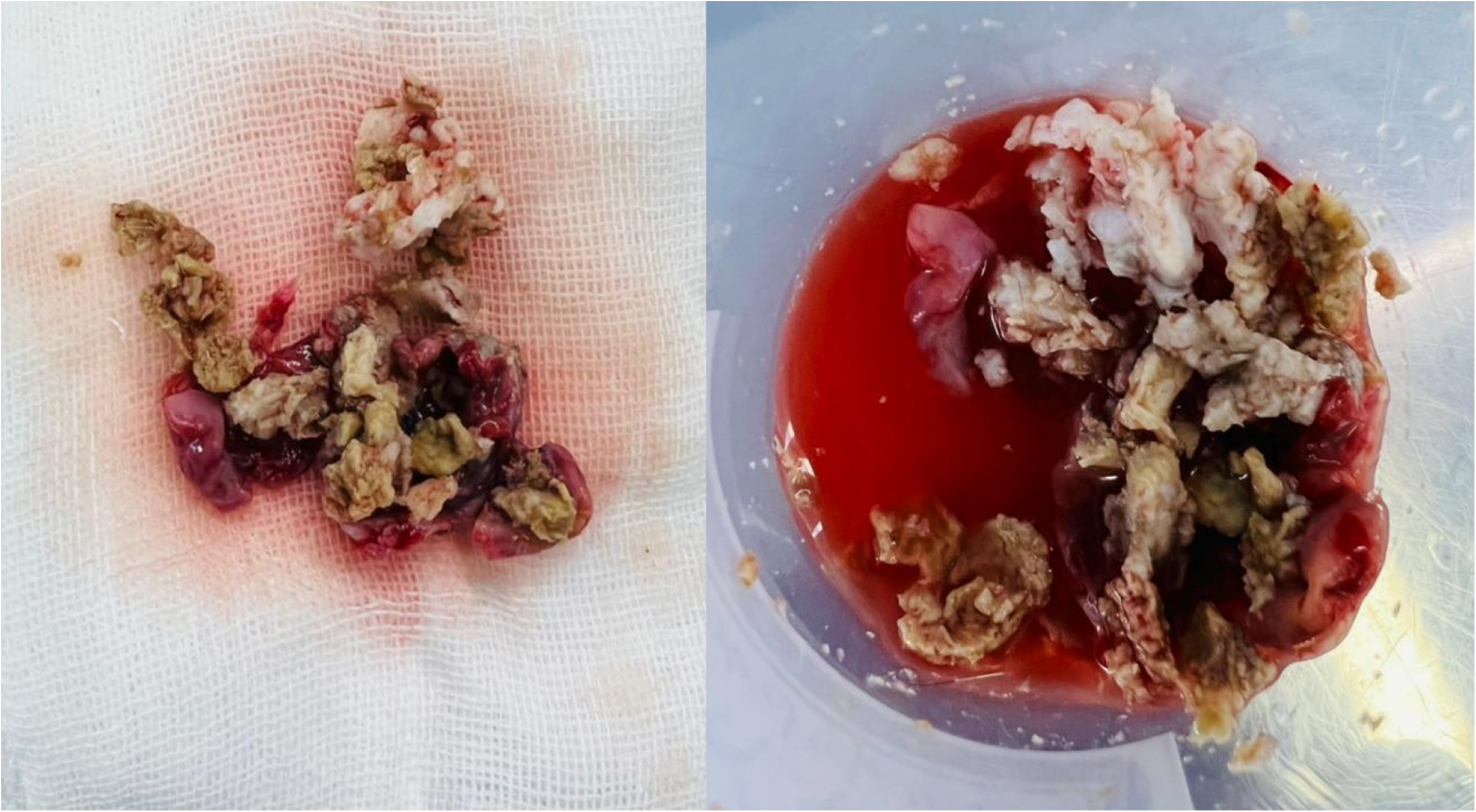
Macroscopic view of the tumoral contents demonstrating hair and fatlike soft tissue.

Magnetic resonance imaging revealed a large‐sized intradural extramedullary capsular lesion at T_11_–L_1_ (conus medullaris) causing spinal cord compression (Figure 1). The patient underwent a surgical operation. A bilateral partial laminectomy was performed from T_11_ to L_1_. Intraoperative findings demonstrated that the thecal sac was relatively swollen; however, the tumor had no extradural invasions. The intradural tumor was positioned in alignment with the conus and between the nerve roots. The tumor capsule was opened under the microscope, and the soft contents inside, including hair and fatlike soft tissue, were removed from the capsule (Figure 2). Furthermore, the adhesions of the capsule around the nerve roots and terminal filum were dissected and separated under microscope magnification. Finally, the tumor was removed entirely along with the capsule.

**FIGURE 3:**
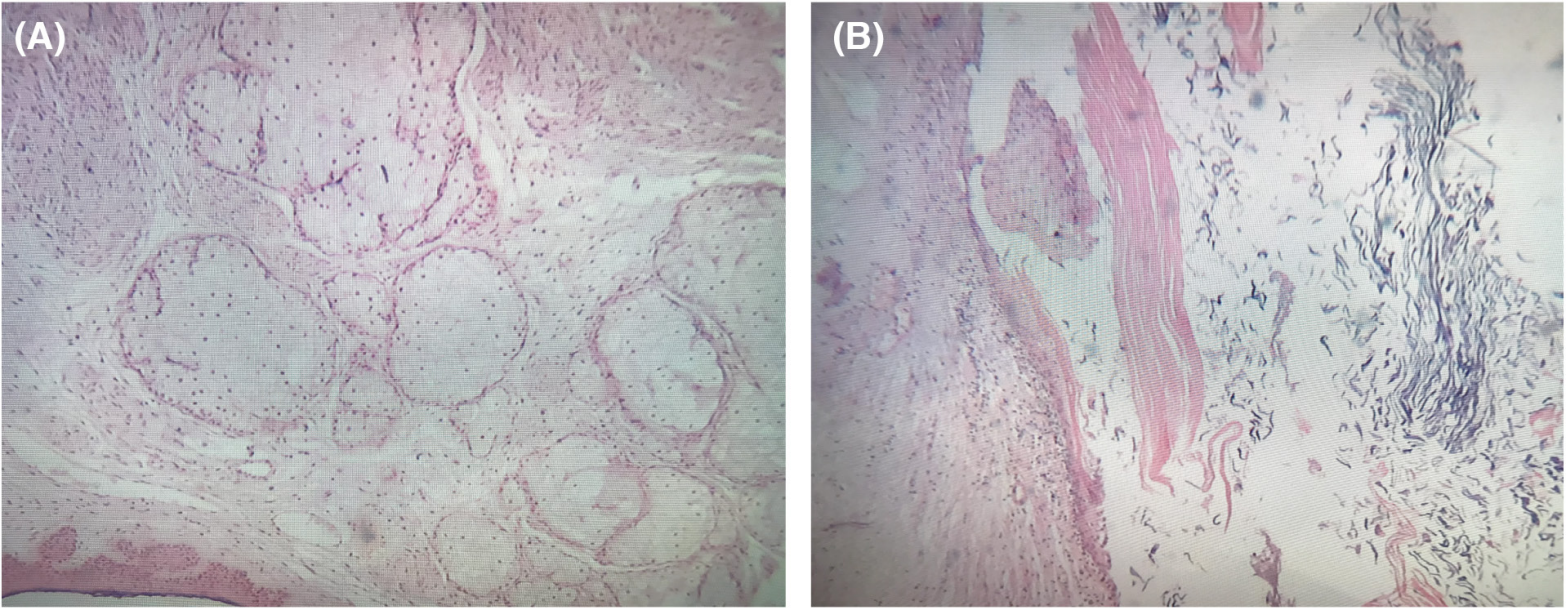
Microscopic histopathological findings demonstrate the presence of central nervous tissue at the periphery and a cyst covered by non‐keratinized stratified squamous epithelium (A). Nervous tissue at the periphery in addition to non‐keratinized stratified squamous epithelium and keratin is shown in (B).

The microscopic investigation of the specimen showed a cystic lesion lined by squamous‐type epithelium and skin adenoma including hair follicles and several sebaceous glands filled with lamellated keratinous materials. Moreover, haphazard nerve bundles as well as low cellular astrogliotic tissue with large ganglion cells were evident. Additionally, some foreign body‐type giant cells, calcification, and chronic inflammatory cell infiltration were also noted, which were suggestive of a ruptured cyst. The findings were consistent with mature teratoma. Figure 3 presents the microscopic view illustrating the histopathological findings.

The patient was discharged with stable hemodynamic conditions 3 days after the surgery. The pain had been significantly alleviated, and the patient did not suffer from any new postsurgical neurological deficits.

## 3 DISCUSSION AND REVIEW OF THE LITERATURE

Teratomas of the CNS are rare, with most cases presenting in the intracranial regions, including the posterior fossa, cerebral hemispheres, pineal gland, and suprasellar region.^3^, ^4^ Compared to intracranial teratomas, teratomas presenting in the spinal cord, particularly the conus medullaris, are exceedingly rare. In the current study, we presented a rare mature teratoma of conus medullaris presenting with prolonged urinary incontinence and progressive radicular pain over time accompanied by foot drop. We also conduct a systematic search of the reports on conus medullaris teratoma and provide an overview of these tumors according to the existing literature.

**FIGURE 4:**
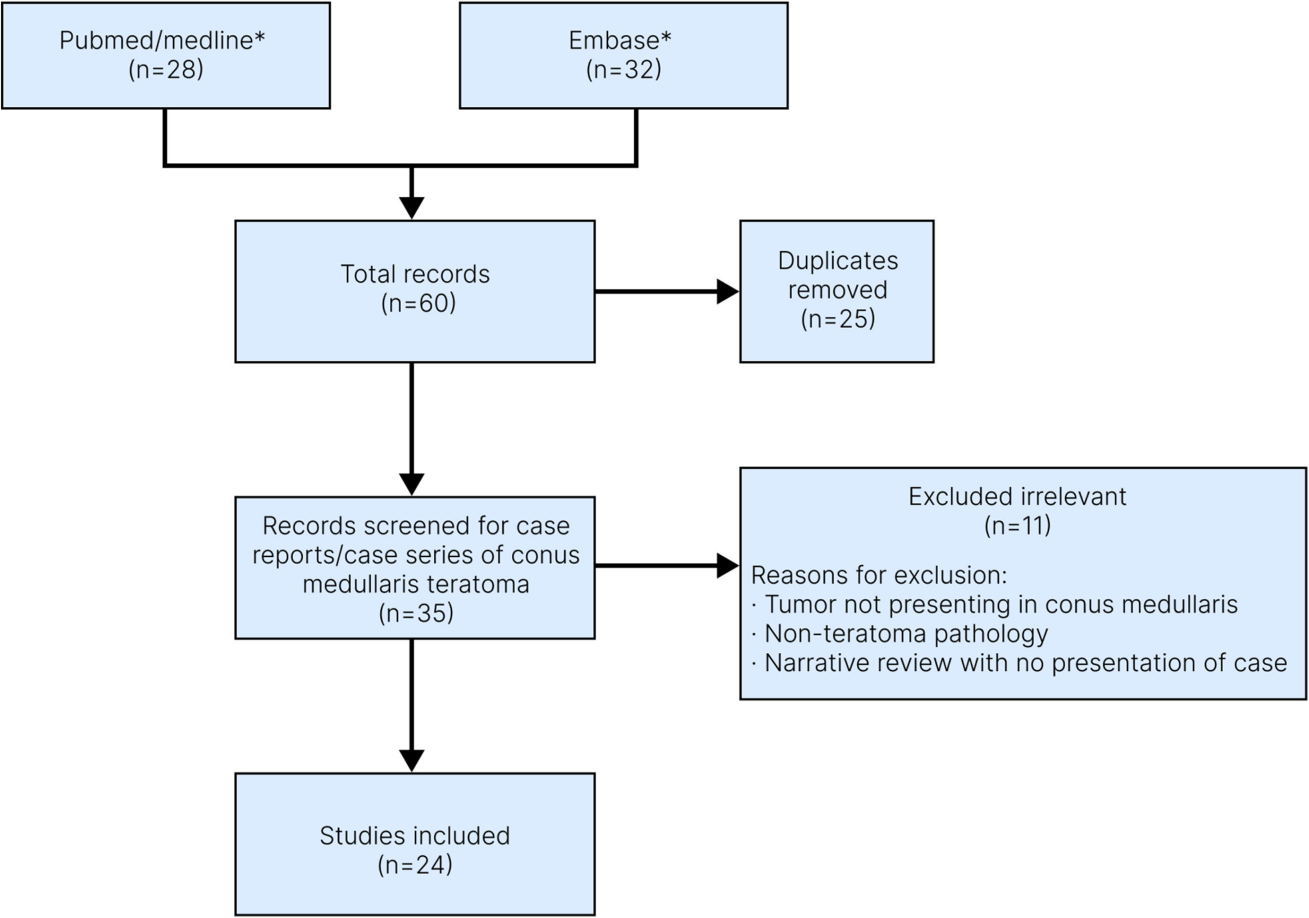
Flow chart of study selection for systematic review of the literature. *Databases were searched for published case reports/case series of conus medullaris teratoma within 2000–2022.

A systematic search of PubMed/Medline and Embase databases was conducted on published case reports and case series of conus medullaris teratoma since the year 2000 (Figure 4). Twenty‐three studies reporting a total number of 63 patients diagnosed with teratoma of the conus medullaris were identified (Table 1). Overall, analyzing the characteristics of the patients reported in the literature demonstrated that the mean age of the patients was 37.1, of which 47.4% were female and 57.6% were male. Lower back pain, radiating lower limb pain, limb numbness, and urinary dysfunction were the most common clinical presentations among patients with teratoma of conus medullaris. Moreover, with lower prevalence, sexual dysfunction had been reported as one of the initial presentations of the tumor in several studies.^5^, ^6^, ^7^ As observed in our case, mature teratoma was the dominant pathologic subtype among all patients in other studies (96.8%), while only two cases of immature teratoma in conus medullaris^8^, ^9^ had been reported.

**TABLE 1:** An overview of the studies reporting cases of conus medullaris teratoma.

Country	Year	Type of report	Age/Mean age	Gender	Pathologic subtype	Tumor extension	Clinical presentation	Reference
Iran	2023	Case report	40	Male	Mature teratoma	T11–L1	Urinary incontinency, low back pain, bilateral lower limb pain and weakness, left foot drop, and saddle hypoesthesia	Present study
Australia	2021	Case report	37	Female	Mature teratoma associated with neuroendocrine tumor	L1–L2	Incidental finding	^31^
India	2021	Case report	25	Male	Mature teratoma	T12–L2	Back pain, bilateral lower limb weakness, and urinary incontinence	^25^
China	2020	Case series of 39 patients	30.9	20 Male and 19 Female	Mature teratoma	T12–S1	Bladder and bowel dysfunction (76.9%), sensory disturbance (72.2%), lower back/leg pain (59.0%), and lower limb weakness (48.7%)	^12^
Iran	2020	Case report	12	Female	Mature teratoma	T12–L1	Back pain and progressive gait disturbance	^32^
Greece	2018	Case report	40	Male	Mature teratoma	L1–L2	Acute lower back pain and left lower extremity numbness and weakness	^33^
Australia	2018	Case report	29	Female	Mature teratoma	L3–L5	Bladder dysfunction, urinary retention, and intermittent lower limb pain	^34^
Iraq	2016	Case report	37	Male	Mature teratoma	L1–L2	Lower back pain, saddle paresthesia, and lower extremity numbness	^35^
Turkey	2016	Case report	12	Male	Mature teratoma	L1–L2	Back pain, bilateral leg weakness, urinary incontinence, and constipation	^26^
USA	2016	Case report	48	Male	Mature teratoma	L2–L3	Muscle fasciculation and cramping of limbs	^15^
Canada	2015	Case report	50	Male	Mature teratoma	L1–L2	Low back pain, bilateral foot numbness, and intermittent bladder hesitancy	^36^
Japan	2013	Case report	42	Female	Mixed germinoma and immature teratoma	L1	Low back pain, buttock numbness, bilateral gluteal and femoral pain	^9^
China	2012	Case report	34	Male	Mature teratoma associated with arteriovenous malformation	L1–L2	Lower back pain, bilateral lower extremity numbness and weakness, and sexual disturbance	^5^
China	2010	Report of two cases	18	Male	Mature teratoma	L2–L4	Low back pain and bladder distention	^37^
			57	Male	Mature teratoma	L1–L2	Back pain and bilateral limb numbness and weakness	
Czech Republic	2009	Case report	52	Female	Mature teratoma	L2–S1	Low back pain, right radicular pain, bilateral limb weakness, and hairy patch in lower lumbar region	^38^
Japan	2009	Case report	68	Female	Mature teratoma	L1–L2	Low back pain, bilateral leg numbness, gait disturbance, urinary dysfunction, and left leg paresthesia	^39^
South Korea	2008	Case report	35	Female	Mature teratoma	T12–L2	Not indicated	^40^
Tunisia	2008	Case report	2	Female	Immature teratoma	Conus medullaris to sacrum	Progressive neurological deficits	^8^
India	2008	Case report	35	Male	Mature teratoma	L3–L5	Intermittent urinary retention and renal failure	^14^
Turkey	2006	Case report	42	Female	Mature teratoma	L1	Back pain and urinary incontinency	^41^
Turkey	2005	Case report	30	Male	Mature teratoma associated with thickened filum terminale	L3–L5 and thickened fatty filum terminale at L5–S1	Low back pain, urinary dysfunction, bladder distention, and erectile and ejaculation dysfunction	^6^
Spain	2004	Case report	46	Male	Mature teratoma	L1–L2	Lumbar pain, bilateral lower limb pain, urinary dysfunction, saddle anesthesia, erectile dysfunction, and fecal incontinence	^7^
Austria	2003	Report of two cases	45	Female	Mature teratoma	T12–L2	Lumbar pain, mild paraparesis, and urinary incontinency	^29^
			20	Male	Mature teratoma	L2–L4	Lumbar pain, lower limb pain, paraparesis, and urinary retention	
Japan	2001	Case report	5	Male	Mature teratoma	Low conus medullaris	Occult spina bifida at birth	^42^

Historically, the first instance of spinal teratoma was documented by Virchow in 1863.^10^ While the exact etiology causing teratomas is unknown, it has been suggested that these tumors arise from primordial germ cells that migrated improperly during embryonic development in the presence of spinal dysraphism. Another prevailing hypothesis on the formation of these tumors suggests that teratomas develop due to the abnormal folding and placement of embryonic cells into the lateral mesoderm.^11^

The clinical manifestations of intraspinal teratomas depend primarily on the tumor's location. Low back pain and lower limb weakness have been reported as the most common symptoms in cases of intraspinal teratomas. The presence of the tumor in the conus medullaris region is associated with other characteristic symptoms, including bladder sphincter or sexual disturbances. For instance, in their study of patients with 39 conus medullaris teratomas, Chen and colleagues reported that bladder and bowel dysfunction were the most common symptoms (76.9%), even more prevalent than lower back/leg pain (59.0%).^12^ Similar to these reports, our case suffered from prolonged urinary incontinence. These findings are foreseeable as the sympathetic innervation of the bladder originates in the lower thoracic and upper lumbar segments of the spinal cord,^13^ and the presence of urinary manifestations might be suggestive of a lesion in the conus medullaris. Additionally, according to our review of the literature, there have been rare cases of conus medullaris teratoma that presented with renal failure at the time of their diagnosis,^14^ suggesting that untreated urinary symptoms might lead to nephrological complications. Moreover, symptoms of intraspinal tumors might vary according to whether the tumor is intramedullary or extramedullary. Intramedullary tumors are rarely known to result in pathognomonic nerve root pain that is more closely linked with extramedullary tumors.^15^ In our case, which was an extramedullary tumor of the conus medullaris, typical nerve root pain was present.

Generally, the most reliable diagnostic tool for spinal teratoma is MRI. The presence of inhomogeneous intensities on T1‐ and T2‐weighted images may indicate tissue heterogeneity, as seen in the solid and cystic components of the tumor, which is suggestive of teratoma. Other imaging techniques, such as plain X‐ray and computed tomography (CT) scans, can also be utilized to detect vertebral anomalies, erosions, calcifications, and increased interpeduncular space.^16^ According to the imaging findings, it might be hard to differentiate spinal teratomas from ependymoma, astrocytoma, and complex neurenteric cysts.^17^ In the present case, MRI findings were also suggestive of ependymoma, while the microscopic description of the specimen was compatible with teratoma. Ependymomas also demonstrate heterogeneous signals on T1‐ and T2‐weighted images. However, the hemorrhagic or necrotic regions seen in ependymomas are uncommon in teratomas.^12^ Overall, histopathologic examination is considered a definitive measure of diagnosis. Accordingly, teratomas are typically diagnosed based on the presence of all three germ layers; however, it has been proposed that determining the total number of layers can be challenging in some instances, as one or two germ layers may grow excessively. Therefore, if a histologic specimen fails to demonstrate all three layers, the diagnosis cannot be ruled out.^18^, ^19^

Teratomas are generally classified into mature, immature, and malignant pathologic subtypes according to their degree of differentiation and the presence of benign or malignant components.^2^ Teratomas that are immature or malignant are characterized by primitive and undifferentiated components and tend to be more aggressive tumors, with a shorter time to metastasis or recurrence.^20^, ^21^ However, these subtypes are less common than their mature counterparts, as our review of the literature revealed only two cases of immature teratoma of conus medullaris compared to 61 cases that had been classified as mature.

Given the uncertain natural progression of teratomas, complete surgical excision is often considered the primary treatment approach.^22^ This is the treatment of choice to prevent progressive worsening of the symptoms, as observed in our patient. Total resection is often utilized to prevent tumor recurrence, but in cases where it may result in neurological impairments or if a firm adherence to the spinal cord parenchyma is present, a subtotal resection might be preferred.^16^ The recurrence rate of teratomas varies based on their histopathological type and the treatment approach. Symptomatic recurrence in mature teratomas, even in cases with subtotal resection, is uncommon. Previous studies have estimated a recurrence rate of about 10%, which is predominantly seen in immature and malignant forms.^23^ Moreover, subtotal resection has been associated with higher recurrence rates than complete resection.^24^ Since these tumors have a slow growth rate, long‐term follow‐up and regular imaging might be necessary. There have been variations in the postoperative outcomes of conus medullaris teratoma, according to previous literature. In our case, which suffered from prolonged symptoms due to lack of timely diagnosis, tumor removal resulted in a significant improvement in low back pain and radicular pain, and the patient did not reveal any postoperative neurological deficits; however, urinary symptoms persisted after the surgery. Similar to our observations, some reports have also indicated that tumor removal resulted in improvement of back pain and leg weakness, while urinary symptoms persisted after the surgery.^25^ In a case series of 39 patients with conus medullaris teratoma, Chen et al.^12^ reported that neurological outcomes were improved in 45.7% of the cases and were stable in 40% of the patients, while in 14.3% these symptoms were aggravated.

Adjuvant radiotherapy is advised for teratomas that include malignant components; however, there is no evidence of the effectiveness of adjuvant chemotherapy in such patients.^26^, ^27^, ^28^ In rare cases of immature teratoma displaying malignant signs, postoperative adjuvant chemotherapy may change the aggressive nature of these tumors and bring about a full and enduring cure.^29^, ^30^ However, more studies regarding the therapeutic value of chemotherapy are needed.

In conclusion, although conus medullaris teratoma is a rare tumor, it can potentially affect patients of any age and cause progressive symptoms, thereby significantly affecting their quality of life. These tumors typically present with a variety of neurological symptoms, including back pain, motor and sensory deficits, and sphincter dysfunction. While the prognosis for these tumors is generally good, there is a risk of symptom progression if proper intervention does not apply. Moreover, in the case of immature or malignant teratoma, there might be cases of recurrence or metastasis.

## AUTHOR CONTRIBUTIONS

**Masoud Eslami:** Conceptualization; data curation; investigation; methodology; supervision; writing – review and editing. **Mehran Ilaghi:** Conceptualization; investigation; methodology; writing – original draft. **Erfan Shahabinejad:** Conceptualization; investigation; methodology; writing – original draft. **Forouzande Khajepour:** Conceptualization; data curation; investigation; methodology; supervision; writing – review and editing. **Saeed Karamouzian:** Conceptualization; investigation; methodology; supervision; writing – review and editing. **Hamed Reihani‐Kermani:** Conceptualization; investigation; methodology; project administration; supervision; writing – review and editing.

## FUNDING INFORMATION

None.

## CONFLICT OF INTEREST STATEMENT

The authors declare that no competing and financial interests exist.

## ETHICS STATEMENT

This study has been conducted according to the guidelines of the ethics committee of Kerman University of Medical Sciences.

## CONSENT

Written informed consent was obtained from the patient to publish this report in accordance with the journal's patient consent policy.

## Data Availability

The data supporting this case report's findings is available from the corresponding author upon request.
